# Using Thin Films of Phase-Change Material for Active Tuning of Terahertz Waves Scattering on Dielectric Cylinders

**DOI:** 10.3390/ma17010260

**Published:** 2024-01-04

**Authors:** Atilla Ozgur Cakmak, Evrim Colak, Andriy E. Serebryannikov

**Affiliations:** 1 School of Engineering, Grand Valley State University, Grand Rapids, MI 49504, USA; 2 Department of Electrical Engineering, Ankara University, Golbasi, 06830 Ankara, Turkey; evrim.colak@ankara.edu.tr; 3 Division of Physics of Nanostructures, Institute of Spintronics and Quantum Information (ISQI), Faculty of Physics, Adam Mickiewicz University, 61-614 Poznan, Poland; andser@amu.edu.pl

**Keywords:** phase-change materials, vanadium dioxide, conductivity, temperature, scattering cross section, Mie resonances

## Abstract

The scattering of electromagnetic waves by isotropic dielectric cylinders can be dramatically modified by means of vanadium dioxide (${\mathrm{VO}}_{2}$) thin-film coatings. Efficient dynamic control of scattering is achieved due to the variations in material parameters realizable by means of external biasing. In this paper, we study the scattering of terahertz waves in a case where the coating shells are made of ${\mathrm{VO}}_{2}$, a phase-change material, whose thin films may work rather as electromagnetic phase screens in the insulator material phase, but as lossy quasi-metallic components in the metallic material phase. The shells that uniformly cover the dielectric cylinders are investigated. Attention will be paid to the demonstration of the potential of ${\mathrm{VO}}_{2}$ in the external control of diverse scattering regimes of the dielectric-${\mathrm{VO}}_{2}$ core–shell scatterer, while conductivity of ${\mathrm{VO}}_{2}$ corresponds to rather insignificant variations in temperature. In line with the purposes of this work, it is shown that the different resonant and nonresonant regimes have different sensitivity to the variations in ${\mathrm{VO}}_{2}$ conductivity. Both the total scattering cross section and field distributions inside and around the core are studied, as well as the angle-dependent scattering cross section.

## 1. Introduction

Phase-change materials (PCMs) represent a wide class of materials whose properties can be dramatically changed by means of biasing [1,2]. PCMs have been extensively used in the last decade in the areas of photonics, advanced electromagnetics, and applied physics. In particular, such thermally tunable materials as ${\mathrm{VO}}_{2}$, GST, and InSb, have been used [3,4,5,6,7,8,9,10,11]. Transition from the metallic (insulator) to the insulator (metallic) phase depends on whether heating or cooling is applied, i.e., higher conductivity corresponds to higher temperature. The attractiveness of PCMs and other tunable materials (e.g., graphene [12] and transparent conducting oxides [13]) is well known, especially in the context of the growing interest in reconfigurable and multifunctional devices working in different frequency ranges [14,15,16,17]. The aforementioned materials can be incorporated in the form of thin uniform layers, resonance enabling or nonresonant inserts to meta-atoms, or like patterned surfaces [8,10,18,19,20,21,22,23,24,25,26,27]. Despite huge progress in nano- and microstructures comprising PCMs, this research area is still far from saturation.

While the components made of phase-change and other tunable materials serve as enablers of a particular functionality of metastructures and relevant applications [28,29,30,31,32,33,34], the studies of individual scatterers of canonical shape represent a separate research direction. Indeed, the aim of the latter is to find the basic features of diverse wave scattering processes, which can be used as input for the design of metastructures for particular applications. Spherical and cylindrical single scatterers or building elements of heterostructures and metasurfaces are often two-component, e.g., they represent core–shell [12,35] or metal–insulator–metal (MIM) [36] structures. It is well known that adding a shell to the original core or metallic components to the original insulator component may completely change the scattering scenario [35,37]. For instance, covering a scatterer with a suitable (uniform or non-uniform) shell may enable superscattering and/or invisibility [38,39,40,41]. Making such scatterer actively tunable or switchable opens a new avenue towards significant reinforcement of functional capability [37,42,43,44]. This remains true regarding the (structures containing) core–shell scatterers with ${\mathrm{VO}}_{2}$ components [45,46,47]. However, tunable materials are unavoidably lossy, which may make a desired functionality impossible, so that the sophisticated ways to their incorporation and, therefore, advanced designs are often needed to mitigate it.

In this paper, we numerically study the scattering of terahertz waves by core–shell cylinders, in which a thin shell is made of ${\mathrm{VO}}_{2}$, while the core is made of Si, an isotropic dielectric material. A big advantage of ${\mathrm{VO}}_{2}$ is that the phase transition is achieved at relatively low temperatures. The main purpose here is to examine the sensitivity of resonant and nonresonant scattering regimes of dielectric cylindrical scatterers to the thermally controlled changes in conductivity of the thin ${\mathrm{VO}}_{2}$ coating shell in a part of the phase transition range, which corresponds to relatively low temperatures and can be achieved rather at insignificant temperature variations. The study is restricted to the cylindrical geometry, one of the canonical geometries of the scatterer, for which analytical solutions for electromagnetic field components can be obtained in terms of the series comprising the special mathematical functions. It will be shown that even a thin ${\mathrm{VO}}_{2}$ shell can totally change the scattering characteristics, as well as the field distribution inside the core. We use a relatively narrow range of variation in ${\mathrm{VO}}_{2}$ conductivity, i.e., it is only varied by a factor of 50, while a much wider range of variation (i.e., by factor of $10^{4}$ and more) has often been considered; see, e.g., Refs. [4,48]. Accordingly, the applied temperature shall be varied just very insignificantly (e.g., by even less than 5 K). This range corresponds to a part of the range of transition from the metallic phase to the insulator phase of ${\mathrm{VO}}_{2}$. Simulations are performed by using conventional Fourier–Bessel series that enable an analytical solution for the amplitudes of spatial harmonics, and thus for near-field and far-field scattering characteristics. The paper is organized as follows. Section 2 presents geometry, material properties and model. Section 3 is dedicated to the thermal tuning of total scattering cross section (SCS). The field distributions in the dielectric core and around it are discussed for different values of ${\mathrm{VO}}_{2}$ in Section 4. The angular effects arising in the far field are investigated in Section 5. Finally, Section 6 presents a brief conclusion.

## 2. Geometry, Materials, and Model

**Figure 1:**
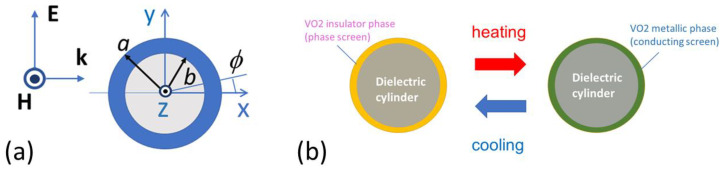
(**a**) General geometry and directions of vectors of electric (**E**) and magnetic (**H**) fields and wavevector (**k**) in case of TE polarization; in case of TM polarization, **E** is along the cylinder axis while **H** lays in (*x*,*y*)-plane; (**b**) schematic demonstrating the principle of thermally tunable scattering.

The studied structure represents a core–shell cylinder, which is infinitely long in the direction perpendicular to (*x*,*y*)-plane; see Figure 1a. The observation angle, ϕ, is measured from *x*-axis in the counter-clockwise direction. The structure is illuminated by a linearly polarized plane wave from the side of negative *x* values, i.e., ϕ=π corresponds to the incidence direction. It is assumed that the core is dielectric, e.g., Si with ε=12, for the sake of definiteness. The shell is made of ${\mathrm{VO}}_{2}$, which is a widely used PCM material showing an insulator–metal–insulator transition [1,49]. The monoclinic-to-tetragonal phase change underlying transition to metallic phase occurs under the increase in temperature, *T*, say, from nearly 330K to 345K [50]. It is known to be reversible but hysteretic while cooling. The values of the complex relative permittivity of ${\mathrm{VO}}_{2}$ strongly depend on crystallographic phase; it varies quickly with *T* in the transition region.

There are different models and data sets for the permittivity and conductivity of ${\mathrm{VO}}_{2}$; see Refs. [51,52,53,54]. For instance, according to Ref. [48], the relative permittivity of ${\mathrm{VO}}_{2}$ can be described in the terahertz range by

(1) $$\varepsilon_{VO2}\left(\omega \right)=\varepsilon_{\infty }-{\left(\omega_{p,VO2}\right)}^{2}/\left[\omega \left(\omega +i/\tau_{VO2}\right)\right],$$

where ω=2πf is angular frequency, *f* is frequency; $\varepsilon_{\infty }=9$, $\omega_{p,VO2}={\left(\sigma_{VO2}/\varepsilon_{0}\tau_{VO2}\right)}^{1/2}$, $\varepsilon_{0}=8.85\times 10^{12}\;F/m$ is free-space permittivity, $\tau_{VO2}=2.27$ fs is relaxation time; conductivity $\sigma_{VO2}=40\;S/m$ for the insulator phase (T=300 K) and $5\times 10^{5}\;S/m$ for the metallic phase (T=400 K). This model has been qualitatively justified by a number of experimental studies, including the recent ones, but there may be some differences regarding the assignment of a given $\sigma_{VO2}$ value to a particular value of *T*. The problem can be even more complicated if we take into account the hysteretic nature of the transition, but this is beyond the scope of this work. In our study, we adopt the model based on Equation (1), in which different values of $\sigma_{VO2}$ are used, but it is not concretized to which *T* values they should correspond. The range of $\sigma_{VO2}$ that is considered in this paper extends from $\sigma_{VO2}=10^{2}\;S/m$ to $\sigma_{VO2}=5\times 10^{3}\;S/m$, so it is expected to be obtainable at small variations in *T*.

Fourier–Bessel series that represent general solutions of Maxwell equations suggest a proper option for this study involving only isotropic materials. The conventional procedure of obtaining amplitudes of spatial harmonics is straightforward (see, e.g., [55]): first, the axial field ($H_{z}$ for TE polarization or $E_{z}$ for TM polarization) is presented as a proper combination of the cylindrical and exponential functions; second, imposing the boundary conditions for the tangential field components in three spatial domains, i.e., r<b, b<r<a, and r>a, yields equations for the unknown amplitudes for all three domains; finally, these equations are solved analytically for the amplitudes. Once they are known, near-zone fields and far-field characteristics can be calculated. Note that the thermal control of scattering by means of thin films of a phase-change material can also be implemented for anisotropic cylinders, although more complex and effort-consuming approaches are needed in this case, e.g., see [56,57,58]. At the same time, a simplified version of one of them [58], which allows one to use a finite-size source of electromagnetic waves instead of a plane wave, can be adapted to an isotropic case.

To quantify scattering in the far zone, the normalized total SCS is used, being given by

(2) $$\sigma_{t}={\left(ka\right)}^{-1}\sum_{m=-\infty }^{\infty }c_{m}c_{m}^{*},$$

where k=ω/c is free-space wavenumber, with *c* being speed of light; $c_{m}$ are amplitudes of spatial harmonics in Fourier–Bessel series at r>a; the asterisk indicates a complex conjugate; ka can be understood as *normalized frequency*. In Equation (2), $\sigma_{t}$ is normalized by SCS of the perfectly conducting cylinder of the radius *a*. This characteristic accumulates contributions from all observation angles, i.e., from 0 to 2π. Also, knowledge of the amplitudes allows us to calculate the ϕ-dependent scattering cross section, $\sigma_{\phi }$.

## 3. Tuning Total Scattering Cross Section

**Figure 2:**
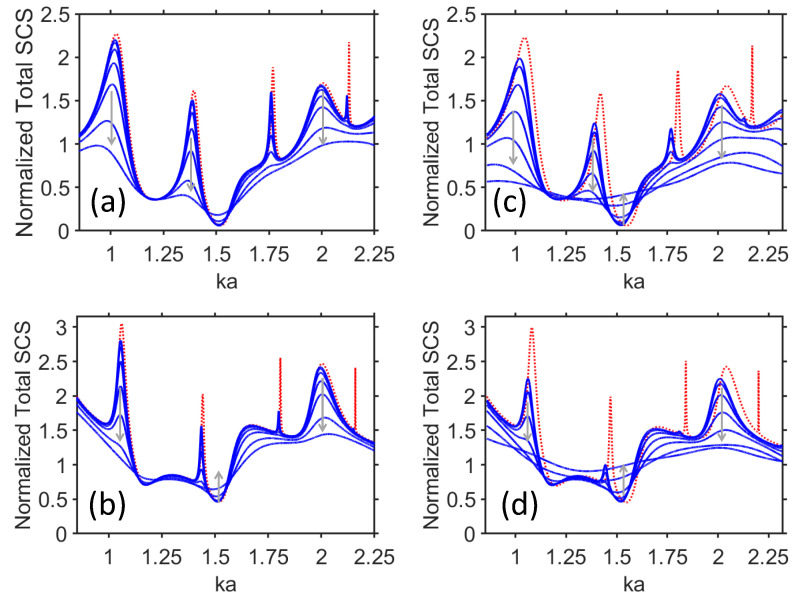
Total SCS, $\sigma_{t}$, as a function of ka, for b=80μm: (**a**) t=0.5μm, TE polarization, (**b**) t=0.5μm, TM polarization, (**c**) t=2μm, TE polarization, (**d**) t=2μm, TM polarization. Conductivity of ${VO}_{2}$, $\sigma_{VO_{2}}$, takes the following values: $10^{2}\;S/m$, $1.4\times 10^{2}\;S/m$, $2.5\times 10^{2}\;S/m$, $5\times 10^{2}\;S/m$, $10^{3}\;S/m$, $2.5\times 10^{3}\;S/m$, $5\times 10^{3}\;S/m$ (blue lines). Arrows indicate the increase in $\sigma_{VO_{2}}$; for comparison, $\sigma_{t}$ is shown in the shell-free case (red line).

In this section, we present the results obtained for $\sigma_{t}$. Figure 2 shows $\sigma_{t}$ as a function of ka, at two selected values of the ${\mathrm{VO}}_{2}$ film thickness, t=a−b, i.e., 0.5μm and 2μm, while b=80μm. For comparison, the results are also presented for the uncoated dielectric cylinder. The values of $\sigma_{VO_{2}}$ are chosen such that they correspond to a part of the material phase transition range, for which temperature is varied insignificantly and kept relatively low, i.e., *T* = 330–340 K.

As observed in Figure 2a,b for both TE and TM polarizations at t=0.5μm, the basic effect arising due to the increase in $\sigma_{VO2}$ is the damping of the scattering maximums, which are mainly connected with volumetric (Mie) resonances [59,60]. The strength of scattering strongly depends on the value of $\sigma_{VO2}$, and hence, on the value of *T*. Existence of the maximums of $\sigma_{t}$ is predetermined by whether the core permittivity is sufficiently large to excite resonances at given sizes. In our case, the maximums occur in a wide range of variation in ka, so that the electrical size of the scatterer is changed from a deep subwavelength to a more than half-wavelength size. Notably, the sharper peaks of $\sigma_{t}$ observed in Figure 2a,b at ka≈1.8 and ka≈2.2 are strongly sensitive to variation in $\sigma_{VO2}$, even if it is relatively small. As observed, a rather strong damping can be achieved, even if adding a shell with $\sigma_{VO2}=10^{2}\;S/m$ to the dielectric core. Generally speaking, the larger Q-factor, the lower the σ-cutoff is, starting from which the $\sigma_{t}$ maximums are almost totally damped.

In turn, here are two scenarios of behavior of $\sigma_{t}$ between the neighboring maximums. In some cases, $\sigma_{t}$ is not changed, regardless of the extent of variation in $\sigma_{VO2}$, as occurs, for instance, at ka=1.2 in Figure 2a and around ka=1.25 in Figure 2b. In the other cases, like the one at ka=1.5 in Figure 2a, a weak scattering (i.e., imperfect invisibility) regime still may be sensitive to the variations in $\sigma_{VO2}$, leading to $\sigma_{t}$ being somehow higher for larger $\sigma_{VO2}$ but remaining low even at small $\sigma_{VO2}$. In fact, there are no significant differences between TE and TM polarizations, except for the aforementioned imperfect invisibility regime, which occurs for the former. In turn, moderately strong scattering is achieved for the respective spectral regime at TE polarization. Notably, the ka values that correspond to the minimums and maximums of $\sigma_{t}$ can be just insignificantly different for the two polarizations. The discussed features allow us to *selectively* control scattering in different parts of the spectrum.

In Figure 2c,d, similar results are presented for t=2μm, i.e., we apply now a four-fold increase in the shell thickness. One can see that the basic features are not changed as compared to the ones in Figure 2a,b. The only significant difference is that the regime of weak visibility, which is observed in Figure 2c for TE polarization at ka=1.5 when $\sigma_{VO2}$ is small, can be switched now for moderate visibility by increasing $\sigma_{VO2}$. Moreover, the first maximum in Figure 2d becomes sharper, and hence more sensitive to variations in $\sigma_{VO2}$, so that there may be a significant difference in $\sigma_{t}$, even when $\sigma_{VO2}$ does not exceed $10^{2}\;S/m$. Next, the sharp resonances observed at ka>2 in Figure 2a, at ka>1.7 in Figure 2b, at ka>1.8 in Figure 2c, and ka>1.4 in Figure 2d either are strongly damped or totally disappear. So, they cannot be gradually tuned in the whole range of $\sigma_{VO2}$ variations, although some kind of on–off switching in the case of sharp resonances is still achievable by using $\sigma_{VO2}=0$ and $\sigma_{VO2}=10^{2}\;S/m$. In other words, the resonance may entirely disappear by a relatively small value of $\sigma_{VO2}$. From the obtained results, it follows that the three main scenarios are possible due to active tuning of the ${\mathrm{VO}}_{2}$ shell: (i) stronger sensitivity at the SCS maximums attributed to volumetric resonances; (ii) weaker sensitivity in the spectral ranges between the maximums; and (iii) on–off switching in the case of very sharp resonances.

As follows from the comparison of Figure 2a,c and Figure 2b,d, sensitivity of $\sigma_{t}$ to the $\sigma_{VO_{2}}$ variations becomes stronger while the ${\mathrm{VO}}_{2}$ shell thickness is increased. However, the sensitivity to the thickness variation strongly depends on whether a resonant or a nonresonant scattering regime is considered. Moreover, for narrower maximums of $\sigma_{t}$, thinner ${\mathrm{VO}}_{2}$ shells are sufficient in order to obtain significant sensitivity of $\sigma_{t}$ to the $\sigma_{VO_{2}}$ variations, whereas the shell should be thicker in case of wider maximums.

**Figure 3:**
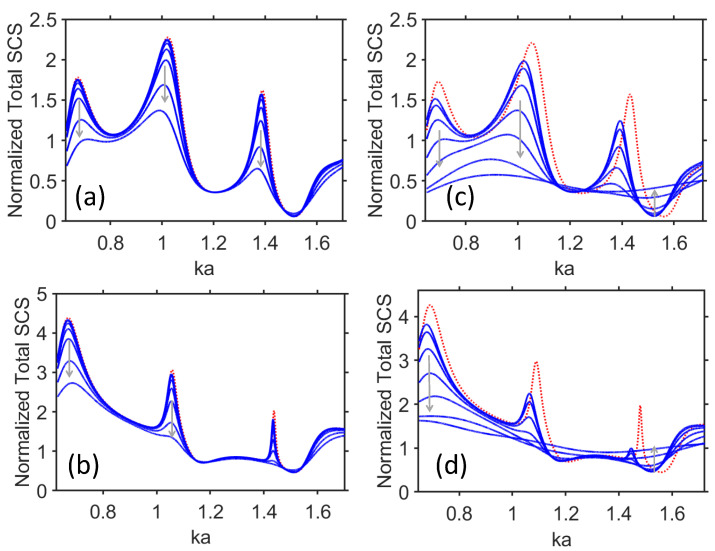
Total SCS, $\sigma_{t}$, as a function of ka, for b=60μm: (**a**) t=0.2μm, TE polarization; (**b**) t=0.2μm, TM polarization; (**c**) t=2μm, TE polarization; (**d**) t=2μm, TM polarization. Conductivity of ${\mathrm{VO}}_{2}$, $\sigma_{VO_{2}}$, takes the values $10^{2}\;S/m$, $1.4\times 10^{2}\;S/m$, $2.5\times 10^{2}\;S/m$, $5\times 10^{2}\;S/m$, $10^{3}\;S/m$, $2.5\times 10^{3}\;S/m$, $5\times 10^{3}\;S/m$ (blue lines). Arrows schematically show the changes of $\sigma_{VO_{2}}$ from smaller to larger values; for comparison, $\sigma_{t}$ is shown in the case without shell (red line).

Figure 3 presents similar results as in Figure 2, but for b=60μm, at the two values of *t*. As observed in Figure 3a,b, there is just an insignificant difference in $\sigma_{t}$ for the cases without a shell and with the shell with $\sigma_{VO2}=10^{2}\;S/m$. The remaining sensitivity-related features are the same as in Figure 2a,b, e.g., the ranges with different sensitivity to the variations in $\sigma_{VO2}$ are alternating. In contrast with Figure 2a, the regime of weak visibility in Figure 3a (at ka≈1.5) is slightly sensitive to the variations in $\sigma_{VO2}$. It may be expected that the significantly larger values of $\sigma_{VO2}$ are needed to switch here for a moderately strong scattering.

One more interesting feature is observed in Figure 3b. The range of weak sensitivity of scattering to the $\sigma_{VO2}$ variations extends from ka≈1.18 to ka≈1.38, while insignificantly varying $\sigma_{t}$. In contrast, the behavior of $\sigma_{t}$ observed in Figure 3c in a wide range of ka variations, i.e., at 0.65<ka<1.1, shows that it is sensitive to the $\sigma_{VO2}$ variations in a wide range. It looks like the best manifestation of the wideband gradual change of $\sigma_{t}$.

Switching between weak and moderately strong visibility is observed in Figure 3c at ka=1.5, similarly to Figure 2c. In the both cases, the shell with a larger $\sigma_{VO2}$ yields higher $\sigma_{t}$ than that with a smaller $\sigma_{VO2}$. This differs from most of the studied regimes in Figure 2 and Figure 3, in which $\sigma_{t}$ is higher when $\sigma_{VO2}$ is smaller. Moreover, as seen in Figure 2d and Figure 3d (for instance, in the vicinity of ka=1.3), $\sigma_{t}$ can be higher for larger $\sigma_{VO2}$, but the weak visibility (imperfect invisibility) regime is not achieved. On the other hand, in Figure 3a,b, the $\sigma_{t}$ values at smaller $\sigma_{VO2}$ exceed the $\sigma_{t}$ values at larger $\sigma_{VO2}$ in the entire ka range, except for the vicinity of ka=1.5, in which $\sigma_{t}$ at larger $\sigma_{VO2}$ is nearly the same but very slightly larger than the one at smaller $\sigma_{VO2}$.

More results obtained by using either Fourier–Bessel series [see Equation (2)] or COMSOL Multiphysics 6.1 commercial software [61] and relevant discussions can be found in Supplementary Material. In particular, Figure S1 presents a numerical simulation environment and an example of extinction efficiency. Figures S2 and S3 illustrate the behavior of a single-scattering albedo, a scattering cross-section, and an absorption cross-section vs. ka, in order to clarify the possible contribution of absorption to the resulting scattering scenarios. Figure S4 presents $\sigma_{t}$ [calculated using Equation (2)] at the continuous variation in the ${\mathrm{VO}}_{2}$ shell thickness, *t*. Finally, Figure S5 presents $\sigma_{t}$ [calculated using Equation (2)] at the continuous variation in $\sigma_{VO2}$.

## 4. Fields in/around the Dielectric-PCM Core–Shell Structure

**Figure 4:**
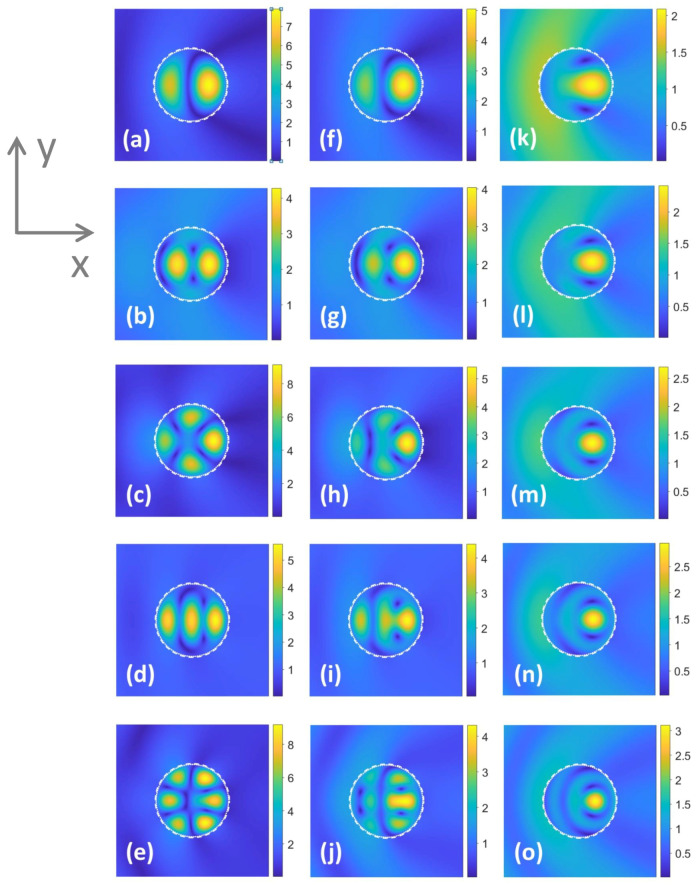
Magnitude of magnetic field, $|H_{z}|$, at (**a**–**e**) $\sigma_{VO2}=10^{2}\;S/m$, (**f**–**j**) $\sigma_{VO2}=10^{3}\;S/m$, (**k**–**o**) $\sigma_{VO2}=5\times 10^{3}\;S/m$; (**a**,**f**,**k**) ka=1.023, (**b**,**g**,**l**) ka=1.21, (**c**,**h**,**m**) ka=1.39, (**d**,**i**,**n**) ka=1.52, (**e**,**j**,**o**) ka=1.768; TE polarization; axes’ directions shown in the inset are the same for all plots from (**a**–**o**). Dashed white circles show location of the shell.

Now, let us consider the axial field distribution in the core and outside it but still in vicinity of the scatterer, for three selected values of $\sigma_{VO2}$ and five selected values of ka. Consideration is restricted to the case of TE polarization (i.e., when the magnetic field is the axial field). The results are presented in Figure 4, so that each of the five rows of the plots corresponds to different values of ka, whereas $\sigma_{VO2}$ is increased from left to right. Accordingly, each of the three columns corresponds to different values of $\sigma_{VO2}$, while ka is increased from top to bottom. The plot rows from top to bottom correspond to the first maximum, first minimum, second maximum, second minimum (case of weak visibility), and third maximum of $\sigma_{t}$, which are observed in Figure 2c. When $\sigma_{VO2}=10^{2}\;S/m$, the dipolar component (|m|=1) is the most contributive component (space component) in Fourier–Bessels series that represent the axial field inside the dielectric core (i.e., $|b_{1}\left|=\max\right|b_{m}|$, where $b_{m}$ stands for the coefficients of the series at r<b), although the pattern shows some asymmetry due to other components of the series (e.g., m=0). The field patterns in Figure 4a,b look similar, but differ in terms of magnitude and extent of asymmetry, so we cannot distinguish between a strong scattering regime and a weak scattering regime while using only field patterns in the core.

Next, in Figure 4c, the quadrupolar component (|m|=2) is dominant. On the contrary, in Figure 4d, the observed pattern results from the joint effect of the different spatial components: first of all, the ones with |m|=0 and |m|=2, which leads to weak visibility (imperfect invisibility). Finally, Figure 4e shows the field distribution at the third maximum of $\sigma_{t}$. Here, the spatial components with |m|=3 are the main contributors. Notably, in Figure 4a–e, just weak asymmetry in the field pattern is observed. This feature is not kept when $\sigma_{VO2}$ is further increased, because the difference between $\max|b_{m}|$ and coefficients for other contributive space harmonics is decreasing. In Figure 4f–j, the results are presented for $\sigma_{VO2}=10^{3}\;S/m$. Similarly to Figure 4a–e, one cannot distinguish between the minimums and maximums of $\sigma_{t}$ based on these results. The general trend observed in Figure 4f–j is the enhancement of the field’s asymmetry, which is attributed to the effects exerted by the currents in the shell. Although the number of spatial maximums for $\sigma_{VO2}=10^{3}\;S/m$ remains the same as for $\sigma_{VO2}=10^{2}\;S/m$, asymmetry manifests itself in such a way that the maximums at the core’s front (i.e., incidence) side are weaker than those at the back (i.e., opposite) side. Notably, intuition says that stronger sensitivity of $\sigma_{t}$ to the variations in $\sigma_{VO2}$ should lead to a stronger change in $\max|b_{m}|$, while just weaker changes should occur when $\sigma_{t}$ is weakly sensitive to $\sigma_{VO2}$ variations. Moreover, in the regime of switching between weak visibility and moderately strong scattering, the coefficients $b_{m}$ are changed dramatically; see Figure 2c, Figure 3c and Figure 4d,j,n.

The further increase in $\sigma_{VO2}$ may yield new features. In Figure 4k–o, the results are presented for $\sigma_{VO2}=5\times 10^{3}\;S/m$. Now, no feature indicates the dominant contribution of the series terms with a particular value of |m|. Indeed, Fourier–Bessel series at r<b have now two [as in Figure 4k] or three [as in Figure 4m–o] comparably contributing space harmonics. All patterns here are asymmetric and show only one pronounced spatial maximum in the core. Interestingly, the number of the pronounced spatial maximums over ϕ is not affected here by the applied ka variations. Based on the obtained results, it is natural to expect that the redistribution of the currents in the shell, being dependent on ϕ and $\sigma_{VO2}$ is crucial for the appearance of the observed field features.

Generally speaking, asymmetry of the field pattern originates from asymmetry in excitation. When $\sigma_{VO2}$ is so small that the shell behaves rather as a phase screen, asymmetry in the field distribution may only originate from the fact that the cylindrical geometry is illuminated by a plane wave (i.e., from a particular side). When $\sigma_{VO2}$ is increased, the shell starts behaving as a conducting screen that changes conditions of excitation of the resonances in the core and serves as the cause of stronger asymmetry. In order to obtain a symmetric field pattern, in both cases, it is necessary to illuminate the structure by a properly chosen number of the incident plane waves whose incidence angles are distributed from 0 to 2π, according to the particular symmetry requirements.

## 5. Angular Effects

**Figure 5:**
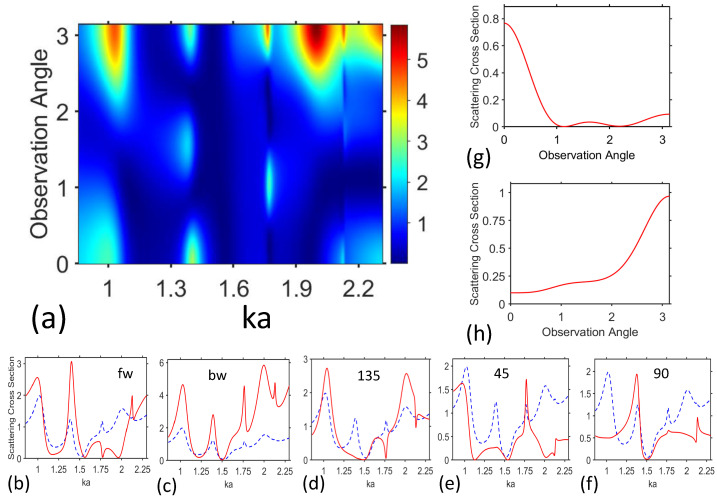
Angle-dependent scattering cross section, $\sigma_{\phi }$ (a.u.), for TE-polarization at $\sigma_{VO_{2}}=10^{2}\;S/m$: (**a**) axial field pattern plotted on (ka,ϕ)-plane; $\sigma_{\phi }$ vs. ka at (**b**) ϕ=0° (forward scattering), (**c**) ϕ=180° (backscattering), (**d**) ϕ=135°, (**e**) ϕ=45° (side scattering), (**f**) ϕ=90°; $\sigma_{\phi }$ vs. ϕ at (**g**) ka=1.48 and (**h**) ka=1.584. In plots (**b**–**f**), $\sigma_{\phi }$ is shown by red solid line; $\sigma_{t}\neq f(\phi )$ is shown by blue dashed line for comparison; observation angle, ϕ, is shown at the ordinate axis in plot (**a**) and at the abscissa axis in plots (**g**,**h**) in units of π; note that the different normalizations are used for $\sigma_{t}$ and $\sigma_{\phi }$, for the sake of simplicity of simulations; arbitrary units used for $\sigma_{\phi }$ are the same in all plots.

In this section, we will investigate the variation of scattering as a function of the observation angle, ϕ. As an example, Figure 5a presents $\sigma_{\phi }$ (in a.u.) on (ka,ϕ)-plane for TE polarization at b=80μm and t=2μm, whereas $\sigma_{VO2}=10^{2}\;S/m$. Backscattering (ϕ=π) is dominant at strong scattering (i.e., when $\sigma_{t}$ is large), but forward scattering (ϕ=0) is also significant. In fact, here we have two basic types of the ϕ-dependence at given ka: (a) both forward scattering and backscattering are significant; (b) only backscattering is significant. From the obtained results, it follows that there are the regions on (ka,ϕ)-plane, in which $\sigma_{\phi }$ does not exceed a particular preset value. Note that although there may be a coincidence between the number of the maximums over ϕ in the field patterns in the core and the number of the maximums of $\sigma_{\phi }$ for some cases, we have rather an unambiguous correspondence between them in the general case.

To further clarify the specifics of contribution of different ϕ-ranges to the overall scattering and specifics of scattering in different ϕ-ranges, Figure 5b–f shows $\sigma_{\phi }$ vs. ka for forward, backward, and three intermediate scattering cases. Obviously, the well-pronounced minimums of $\sigma_{t}$ as a function of ka mean that the minimums of $\sigma_{\phi }$ are expected to appear for all values of ϕ. In contrast, quite strong maximums of $\sigma_{\phi }$ may appear even if $\sigma_{t}$ is low within a particular part of the entire ϕ range. As observed, the maximum of $\sigma_{\phi }$ occurs at ka=1.023 for both forward (ϕ=0°) and backward (ϕ=180°) scattering, and for ϕ=135°, but it is rather weak at 45°, and 90°, see Figure 5b–f. This type of far-field behavior correlates well with the main contribution of the components with |m|=1 in the near field; see Figure 4a. In turn, for the maximum at ka=1.39, scattering is strong at ϕ=0°, 90° and 180°, but weak at ϕ=45° and 135°; it agrees with the main contribution of the components with |m|=2 to the field distribution in the core, as observed in Figure 4c. It is noteworthy that for the both ka=1.023 and ka=1.39, the number of the maximums of $\sigma_{\phi }$ over ϕ (*p*) in Figure 5a–f is equal to the number of the maximums of field magnitude in the core (*q*) in Figure 4a,c. Next, at ka≈1.77, $\sigma_{\phi }$ is high at ϕ=45° and 180°, but tends to zero at ϕ=0° and 135°. In this case, we observe that p≠q, i.e., $\sigma_{\phi }$ is near zero in vicinity of the particular ϕ values, at which the axial field inside the core has the maximum. Finally, at ka≈2, we observe the maximums of $\sigma_{\phi }$ at ϕ=135° and 180°, but weak or moderately high $\sigma_{\phi }$ at other angles, which indicates the main role of backscattering in the overall scattering process.

Figure 5g,h presents $\sigma_{\phi }$ as a function of ϕ for the selected values of ka, with the purpose of demonstrating that either forward or backward scattering can dominate for the two close and carefully adjusted ka values. This happens due to the fact that the regions of high $\sigma_{\phi }$, which are observed in Figure 5a for the same resonance regimes but at different ϕ, may show different widths and are somehow shifted with respect to each other in terms of ka. In fact, we observe in Figure 5g the forward scattering features that are associated with Kerker effect, but in our case, the region of backscattering is rather shifted than suppressed, in contrast to what is observed when the Kerker conditions are fulfilled; see [62,63,64]. The forward scattering is achieved here at the price of relatively low efficiency. It is worth noting that the scattering features observed in Figure 5g and those in Figure 5h are attributed to different resonances. At the same time, similar shifts are observed in Figure 5a in the vicinity of ka=1 and in the vicinity of ka=1.39.

**Figure 6:**
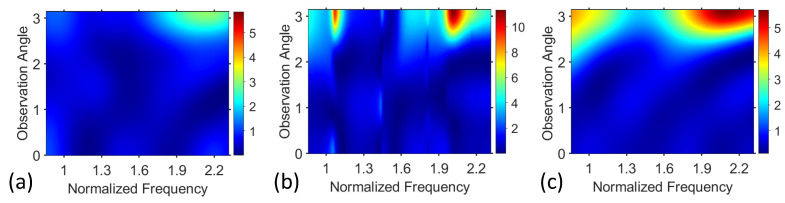
Angle-dependent scattering cross section, $\sigma_{\phi }$ plotted on (ka,ϕ)-plane for (**a**) TE-polarization at $\sigma_{VO_{2}}=2.5\times 10^{3}\;S/m$; (**b**) TM-polarization at $\sigma_{VO_{2}}=10^{2}\;S/m$; (**c**) TM-polarization at $\sigma_{VO_{2}}=2.5\times 10^{3}\;S/m$; normalized frequency has units of ka.

While $\sigma_{VO2}$ is growing, $\sigma_{t}$ as a function of ka is smoothing and resonances are becoming weaker pronounced (see Figure 2 and Figure 3). At the same time, the field pattern is significantly modified (see Figure 4). Figure 6a shows $\sigma_{\phi }$ on the (ka,ϕ)-plane for TE polarization when $\sigma =2.5\times 10^{3}\;S/m$ is taken. We can see that sharp (narrowband) features like the ones observed in Figure 5a do not appear herein. Strong and wideband backscattering takes place at 1.9<ka<2.3, but forward scattering is also not vanishing, and in some cases can be said to be moderate.

For the sake of completeness, Figure 6b,c show $\sigma_{\phi }$ on the (ka,ϕ)-plane for TM polarization, at $\sigma_{VO2}=10^{2}\;S/m$ and $\sigma_{VO2}=2.5\times 10^{3}\;S/m$, respectively. In Figure 6b, we can see that the basic features are similar as the ones for TE polarization. In particular, it is observed that backscattering dominates in the overall scattering; ka ranges with the dominant backscattering and with the dominant forward scattering can be slightly shifted along the ka axis, so that the forward scattering can be dominant. Notably, the patterns in Figure 5a and Figure 6b shown for $\sigma_{VO2}=10^{2}\;S/m$ are rather similar to those at $\sigma_{VO2}=0$, but here, we are interested in small but nonzero values, because they can be a part of the tunable scenarios. The field pattern presented in Figure 6c shows similar features as the ones in Figure 6a in the case of TM polarization. The only principal difference is that $\sigma_{\phi }$ in Figure 6c is also rather large at ka<1, not only around ka=2.

## 6. Conclusions

In this paper, we numerically studied the effects of the thermally biased conductivity of vanadium dioxide (${\mathrm{VO}}_{2}$) on scattering by core–shell (here, dielectric-${\mathrm{VO}}_{2}$) cylindrical structures. The results were obtained for the both far- and near-field characteristics. The conductivity of ${\mathrm{VO}}_{2}$, $\sigma_{VO2}$, was varied in a relatively narrow range, i.e., from $10^{2}\;S/m$ to $5\times 10^{3}\;S/m$, but it is shown to be sufficient for enabling dramatic changes in the scattering. Strong sensitivity of scattering to the variations in $\sigma_{VO2}\left(T\right)$ has been observed, even if the thickness of the ${\mathrm{VO}}_{2}$ shell is small compared to the core diameter, which is less than the free-space wavelength within the considered frequency range.

By choosing sizes of the core and shell, we can achieve a particular (set of) tuning/switching scenario(s). Different sensitivity to the ${\mathrm{VO}}_{2}$ variations is achieved for different volumetric resonances in the core, so that the sharper the maximum (i.e., higher Q-factor), the stronger the sensitivity to variations of $\sigma_{VO2}$. Along with the regimes of strong and weak (or even vanishing) sensitivity, which appear, respectively, at and between the resonance maximums, and which are discussed in detail in this paper, it is worth mentioning that adding even a weak but nonzero conductivity results in the suppression of the most sharp (highest-Q) maximums of the scattering cross section, as occurs, for instance, when changing the conductivity from very small values to $10^{2}\;S/m$. Therefore, only the range of small $\sigma_{VO2}$ is suitable for obtaining tunable scattering scenarios in this case. Also, switching between weak visibility (imperfect invisibility) and moderate scattering can be achieved.

From the obtained results, it follows that the regimes of weak/moderate and strong scattering cannot be distinguished, based on the near-field patterns, because they differ rather by the field magnitude. Generally, an increase in $\sigma_{VO2}$ leads to both the field distribution in the core and the total scattering cross section being changed significantly. In particular, the extent of asymmetry of the former with respect to the scatterer mid-plane (i.e., the plane perpendicular to the incidence direction) is increasing with $\sigma_{VO2}$.

Angular dependence of the scattering cross section is shown to be strongly sensitive to the variations in $\sigma_{VO2}$ with *T*. At $\sigma_{VO2}=10^{2}\;S/m$, it is found the the angular dependence of the scattering cross section is very coherent for lower-order resonances with the features observed in the field distributions inside the core. However, additional consideration is needed for higher-order resonances. The differences in the scattering scenarios at $\sigma_{VO2}\propto 10^{2}\;S/m$ and $\sigma_{VO2}\propto 10^{3}\;S/m$ are clearly indicated. The obtained results further demonstrate the perspectiveness of PCMs in active control of scattering. As the next steps of this research program, it is planned to study the specifics of tunable scattering on the partially coated cylinders, adjust the temperature range required to obtain the targeted range of $\sigma_{VO2}$, study scattering under a hysteretic behavior of ${\mathrm{VO}}_{2}$, and introduce optical switching by means of carrier generation in PCM film for both two- and three-dimensional structures.

## Data Availability

Data underlying the results presented in this paper are not publicly available, but can partially be obtained from the corresponding author upon a reasonable request.

## Supplementary Materials

The following supporting information can be downloaded at: https://www.mdpi.com/article/10.3390/ma17010260/s1, Figure S1: Depiction of the Numerical Simulation Environment and an exemplary Extinction Efficiency plot for different conductivities for TE illumination. Figure S2: TE polarization incident on the cases investigated in Figure 2c. Presenting Single Scattering Albedo, Absorption and Scattering Cross Section values for three distinct conductivity values. Figure S3: TM polarization incident on the cases investigated in Figure 2d. Presenting Single Scattering Albedo, Absorption and Scattering Cross Section for three distinct conductivity values. Figure S4: TE Normalized SCS values for the scanned ${\mathrm{VO}}_{2}$ thicknesses plotted for different frequencies and for three distinct ${\mathrm{VO}}_{2}$ conductivity values. Figure S5: TE Normalized SCS values for the scanned ${\mathrm{VO}}_{2}$ conductivities plotted for different frequencies and for two distinct ${\mathrm{VO}}_{2}$ thicknesses.

## Abbreviations

The following abbreviations are used in this manuscript:

MIM	Metal–Insulator–Metal
PCM	Phase-Change Material
Q-factor	Quality factor
SCS	Scattering Cross-Section
