# Supplementary material for: Using Thin Films of Phase-Change Material for Active Tuning of Terahertz Waves Scattering on Dielectric Cylinders
Source: Materials (Basel). 2024 Jan 4;17(1):260. doi: 10.3390/ma17010260 (PMC10780087; doi:10.3390/ma17010260)
Supplement: Supplementary file 1 [file materials-17-00260-s001.zip › materials-2716260-supplementary.pdf]

Supplementary Materials

Using thin films of phase-change material for active tuning of terahertz waves scattering on dielectric cylinders

Atilla Ozgur Cakmak[1,†*], Evrim Colak[2,†], and Andriy E. Serebryannkiov[3,†]

[1]School of Engineering, Grand Valley State University, Grand Rapids, Michigan, USA; cakmaka@gvsu.edu

[2]Department of Electrical Engineering, Ankara University, Golbasi, 06830 Ankara, Turkey; Evrim.Colak@ankara.edu.tr

[3]Division of Physics of Nanostructures, ISQI, Faculty of Physics, Adam Mickiewicz University, 61-614 Poznan, Poland; andser@amu.edu.pl

***Correspondance: cakmaka@gvsu.edu**

†These authors contributed equally to this work.

1- Introduction of the Numerical Simulations and useful terminologies:

A numerical simulation setup has also been realized on top of the available analytical solutions addressed in the paper. As the incoming wave propagates along the +x direction (as shown in Fig. S1(a)), the extinction efficiency (EE) has been calculated. The dielectric cylinder is surrounded by the VO_2 shell and the simulation domain is encapsulated with phase matching layers (PML) in a two-dimensional (2-D) configuration. The radius of the simulation environment (r_{PML}) together with the PML thickness (t_{PML}) have been optimized to reduce the artificial reflection back into the simulation domain together with the mesh size. Accordingly, the input power given to the system is given in Eq. S1 in units of W/m^2.

$$\boldsymbol{P_{inc}} = \frac{\mathbf{1}}{\mathbf{2}\boldsymbol{Z_0}} |\boldsymbol{E_{inc}}|^2 \hat{\boldsymbol{k}} \tag{S1}$$

where $\hat{\boldsymbol{k}}$ is the propagation direction of the Poynting vector and $\boldsymbol{Z_0}$ is the free space wave impedance. Then, scattered power ($\boldsymbol{W_{sca}}$) is found out with a line integral mimicking a surface integral in its 3D counterpart over the cylinder surface in units of W/m. Simulation setup solves for the scattering fields ($\boldsymbol{E_{sca}}, \boldsymbol{H^*_{sca}}$) as they can be individually found out by decomposing the total field into scattered and incident waves in Maxwell's equations.

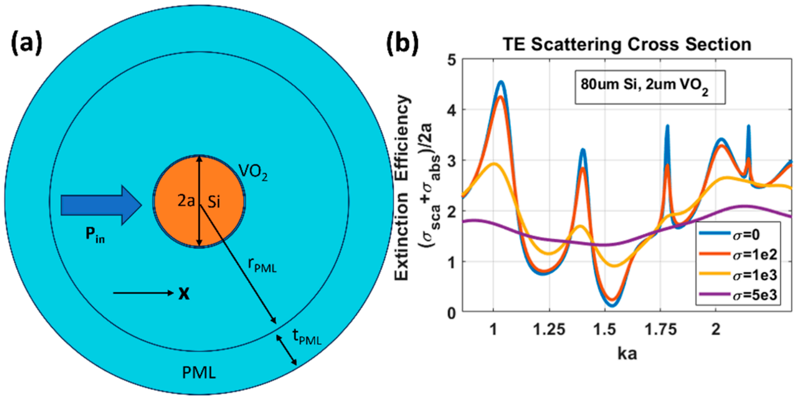

Figure S1: (a) Numerical Simulation Environment, (b) EE vs. ka for different conductivities of VO_2 ranging from 0 to 5e3 S/m.

$$\boldsymbol{W_{sca}} = \frac{\mathbf{1}}{\mathbf{2}} \oint_{\boldsymbol{2\pi a}} \boldsymbol{Re\{E_{sca} \times H^{*}_{sca}\} \cdot \hat{n} dl} \tag{S2}$$

Eq. S2 shows $\boldsymbol{W_{sca}}$ for this 2-D system while $\boldsymbol{\hat{n}}$ is normal to the tangent line at every point of the cylinder surface/line along $\boldsymbol{dl}$. Likewise, the absorbed power by the cylinder can be found out by evaluating an area integral over the full dielectric core+shell as given in Eq. S3 in units of W/m.

$$\boldsymbol{W_{abs}} = \iint_{\boldsymbol{\pi a^2}} \boldsymbol{Q_{loss}} ds \tag{S3}$$

where $\boldsymbol{Q_{loss}}$ is the energy loss rate specifically within the VO_2 layer due to the increasing conductivity values with the slightly elevated temperature. Hence, the cross-section efficiencies (unitless) are found out in the canonical form of:

$$\boldsymbol{\sigma_{sca}} = \frac{\boldsymbol{W_{sca}}}{\boldsymbol{P_{in}}}, \boldsymbol{\sigma_{abs}} = \frac{\boldsymbol{W_{abs}}}{\boldsymbol{P_{in}}}, \frac{\boldsymbol{\sigma_{sca}}}{\boldsymbol{2a}} + \frac{\boldsymbol{\sigma_{abs}}}{\boldsymbol{2a}} = \boldsymbol{Q_{sca}} + \boldsymbol{Q_{abs}} = \boldsymbol{Q_{ext}} \tag{S4}$$

where 2a happens to be the cross section in this 2-D configuration and $\boldsymbol{Q_{sca}}$, $\boldsymbol{Q_{abs}}$ and $\boldsymbol{Q_{ext}}$ indicate the scattering, absorption, and extinction efficiencies, respectively. One last figure of merit, which is single-scattering albedo (SSA), has also been investigated in its canonical form of:

$$\boldsymbol{\omega} = \frac{\boldsymbol{\sigma_{sca}}}{\boldsymbol{\sigma_{ext}}} \tag{S5}$$

as $\boldsymbol{\sigma_{ext}}$ indicates the extinction cross section and $\boldsymbol{\omega}$ becomes a useful factor in analyzing the fraction of the scattering to the absorption losses in the discussion of invisibility. As $\boldsymbol{\omega}$ is getting close to 1 (scattering dominant), if the scattering mechanisms are also lessened at the same time, we may be able to talk about invisibility.

2- Discussion of the influence of the absorption on scattering mechanisms of the VO_2 coated cylinders:

It should be emphasized that the discussed scattering mechanisms in the main manuscript are strictly related to the relatively weak tuning of the conduction of the VO_2 shell with slight temperature changes. Hence, the main text rigorously considers normalized scattering cross section (SCS) only. However, there are loss mechanisms stemming from the introduction of the conductivity inherent to the VO_2 shell. *We would like to investigate the effect of the absorption mechanisms for completeness in the supplementary section to make sure that the focus of the manuscript is still constructed around the normalized SCS.* We have obtained the contributions of the loss mechanisms via using numerical methods instead of utilizing Fourier-Bessel series. Figures S2 and S3 portray the calculation of the corresponding TE and TM cases given in Fig. 2(c) and (d) in the present paper with t = 2 μm thickness of a shell and a = 82 μm.

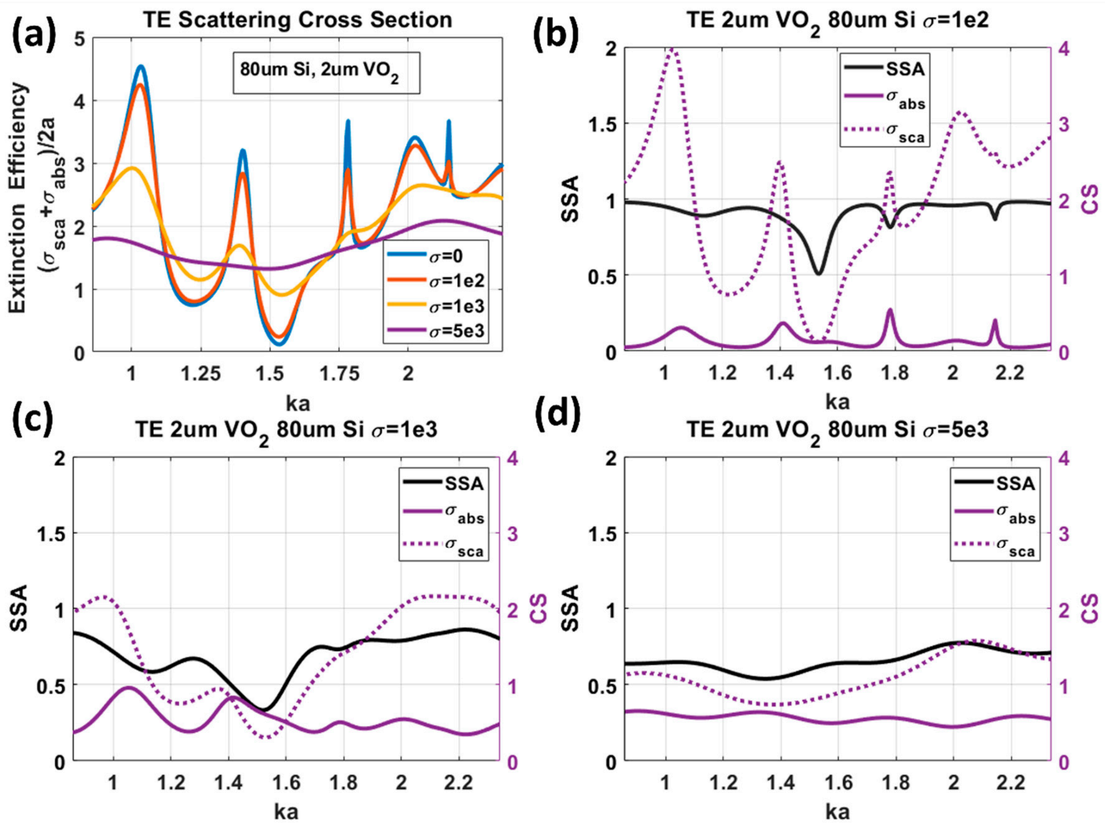

Figure S2: TE polarization incident on the cases investigated in Fig. 2(c): t = 2 μm and a = 82 μm. (a) EE vs. ka values for increasing VO_2 conductivities. SSA vs ka values - left axis and Cross-section (CS) – right axis in (b)-(d). Absorption CS – solid and Scattering CS – dotted curves. Calculated for σ_{VO2} (b) 1e2 S/m, (c) 1e3 S/m, and (d) 5e3 S/m.

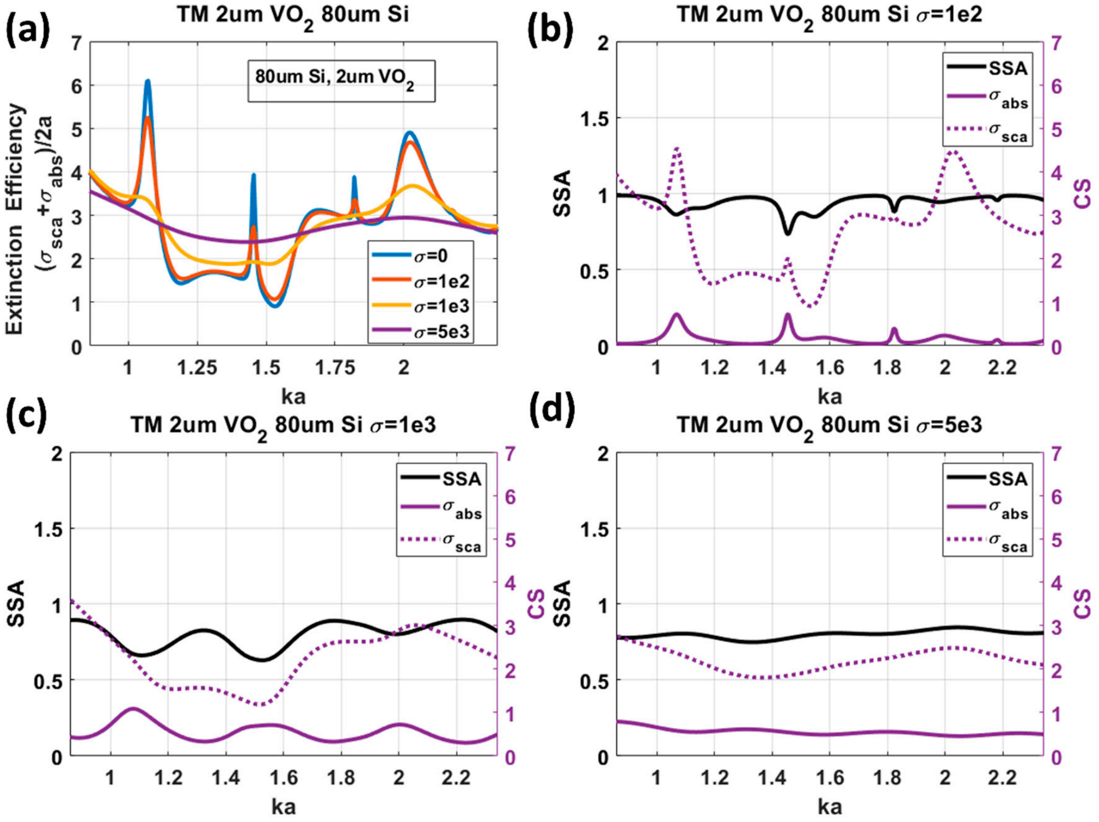

Figure S3: TM polarization incident on the cases investigated in Fig. 2(d): t = 2 μm and a = 82 μm. (a) EE vs. ka values for increasing VO_2 conductivities. SSA vs ka values - left axis and CS – right axis in (b)-(d). Absorption CS – solid and Scattering CS – dotted curves. Calculated for σ_{VO2} (b) 1e2 S/m, (c) 1e3 S/m, and (d) 5e3 S/m.

If we summarize the main outcomes Figs. S2-3 in conjunction with Fig. 2(c) and (d):

1) Main features of dampening the scattering CS are *still* at work in both Figs. S2(a) and S3(a) with the increasing conductivity.
2) Sharp resonances are quickly affected and even destroyed by the increasing conductivity *in agreement* with Figs. 2(c) and (d).
3) TM peaks are *still* narrower than TE peaks.
4) The first difference stemming from the absorption in the EE spectra is the remodification of the band around ka=1.25, which used to be rather flat previously and insensitive to σ_{VO2} changes in Fig. 2(c). Now, obviously the absorption boosts the EE for higher σ_{VO2} values, as expected.
5) As for the weak invisibility region around ka=1.5 in TE case, we can talk about SSA values dropping down to 0.5 in Fig. S2(b), which in other words signify that regardless of the dampening of the SCS, the complete invisibility would be spoiled

by the losses. However, within the context of the weak invisibility, similar conclusions mentioned in the main paper can be reached as SCS is already quite small.

6) SSA values indicate that the loss mechanisms' contribution on the total CS become more pronounced for higher σ_{VO2} values together with the dampening effect of the scattering. The interactions between the cylinder and the incoming wave once predominantly (higher than 90%) ruled by the scattering for low σ_{VO2} values start to feel the implications of the loss mechanisms as the weight of the losses increase up to roughly 20-30% range in Figs. S2(d) and 3(d).
7) SSA values are always higher than > 50% for all cases and ka values apart from the brief drop around the narrow band of ka=1.5, namely the weak scattering region, in Fig. S2(c).
8) The absorption bands also become broader with the increasing σ_{VO2} values in general just as in the case of the volumetric sharp resonance bands, as expected.

In summary, absorption bands contribute to the effective cross section. Yet, complete invisibility or cloaking do not constitute the major components of the present work. Therefore, absorption does not critically jeopardize the main functionalities of a tunable device, which is introduced in the main text.

3- Discussion of the influence of the shell thickness on scattering mechanisms of the VO_2 coated cylinders:

Analyses have been directed towards the elucidation of the effect of the chosen shell thickness in the scattering mechanism. Figures 2(a)-(c) and (b)-(d) had already shown a 4-fold increment comparatively (switching from a shell thickness of t = 0.5 μm to 2 μm) and its outcomes on the scattering and tunability had been reported in the present work. Accordingly, the scattering tunability may become very sensitive for thicker shells such that a very tiny σ_{VO2} change could alter the scattering mechanism. On the other hand, less sensitivity at smaller thicknesses might yield an opportunity for better control on the tunability with the altered temperature. Further scans have been carried out with the aid of Fourier-Bessel functions based analytical methods to clarify the sensitivity of the scattering on thickness changes. The results have been plotted in Fig. S4. The respective σ_{VO2} increment is from 1e2 S/m to 5e3 S/m (x50) from (a) to (c). Four bands have been selected from Fig. 2, which are corresponding to the 1st maximum (ka=1.021), 1st minimum (ka=1.205), 2nd maximum (ka=1.385) and 3rd maximum (ka=1.76) while $t = a\text{-}b$ by definition. The two selected thickness values (t = 0.5 and 2 μm) are shown with dashed lines for guidance on the plots. It is apparent that t = 0.5 μm enables better control of the

total normalized SCS as it is less sensitive. Nonetheless, in the cases of applications where only very minor changes in the temperature are foreseen, larger thicknesses might be more appropriate due to their increased sensitivity. It could also be deduced that most of the monotonous tunability effects are occurring till $t \leq 2$ μm for all resonance-involved cases where SCS is dropping with increased σ_{VO2}. We may encounter less sensitivity against σ_{VO2} tunability and even slight increments in SCS after $t = 3$ μm. Hence, the studied region in the main manuscript promises an easier, predictable tunability for subsequent applications. It can be observed that the green band, which is the 1st minimum (ka=1.205) happens to be rather flat and insensitive to the thickness changes as it is not governed by the volumetric resonance scattering.

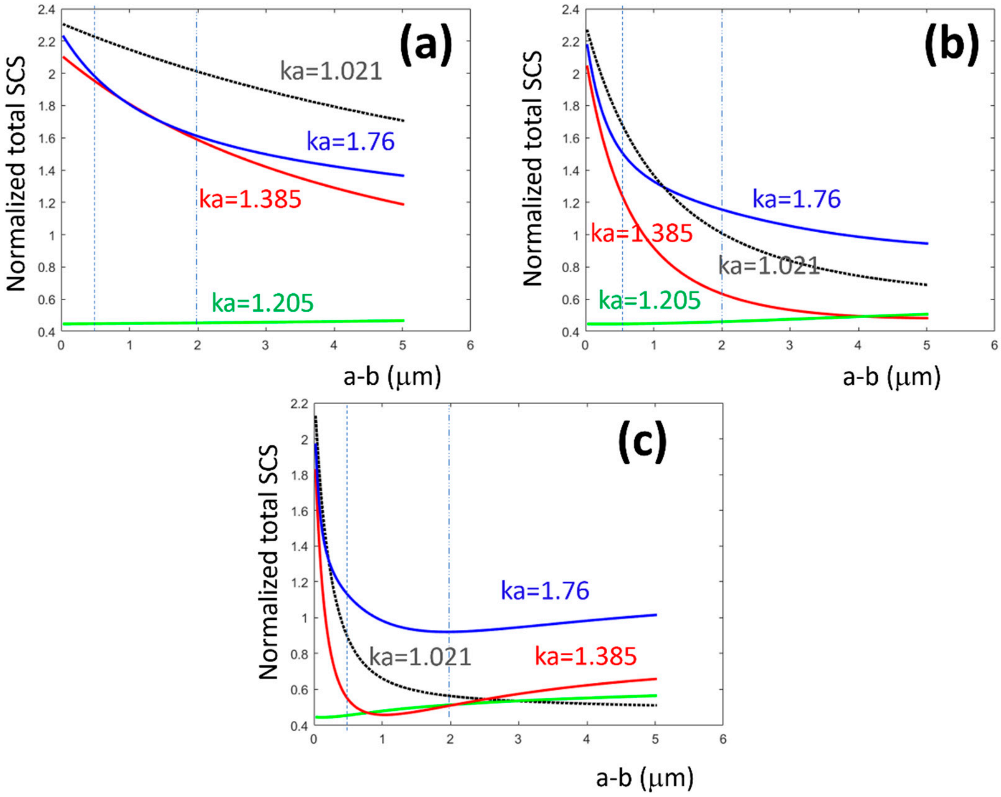

Figure S4: TE Normalized SCS values plotted with the scanned t = a-b for several frequency points that are selected from various minimum and maximum bands in Fig. 2. The corresponding σ_{VO2} values are (a) 1e2 S/m, (b) 1e3 S/m and (c) 5e3 S/m.

As the relatively broader ka=1.021 band (lower quality factor, some immunity against absorption) is governed by the scattering effects as opposed to the losses, signatures of monotonous tunability is evident as σ_{VO2} is increasing even at thicknesses as high as $t = 5$ μm. Yet, the sensitivity is dropping for such thicker shells compared to the ones around

t = 2 μm. On the other hand, sharper resonances in ka=1.385 and ka=1.76 bands are prone to losses. They offer a somewhat similar tunability up to around *t* = 3 μm where the respective monotonous drop changes its course for the benefit of slight increments. As a result, thicker shell cases involve loss mechanisms that present clues for us in terms of their present difficulty in adaptation as a device. Keeping in mind that adding extra thicknesses is effectively akin to increasing the σ_{VO2} values, thinner shells that are less than 2 μm present themselves as rather promising candidates suitable for easier tunability.

4- Discussion of the influence of the selected σ_{VO2} tunability range on scattering mechanisms of the VO_2 coated cylinders:

Finally, an investigation has been carried out to shed light on the availability of the conductivity-based tuning of the SCS with the presented design. The same bands of operation have been picked from Fig. 2, which were previously used in the discussions concerning the thickness effects, too. As Fig. S5 clearly demonstrates, SCS is very easily tuned with relatively small changes in the conductivity. An intriguing effort could be to come up with a linear regression-fit curve that relates σ_{VO2} directly to the attained SCS.

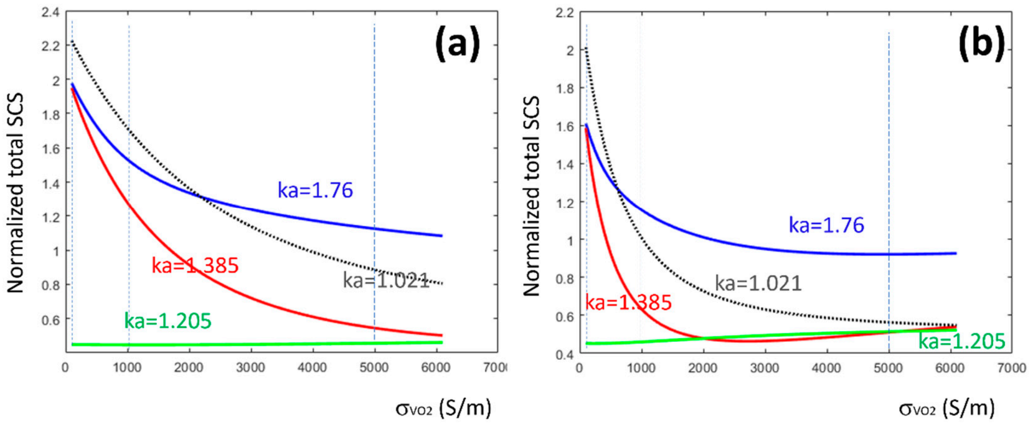

Figure S5: TE Normalized SCS values plotted while scanning σ_{VO2}. Same max. and min. bands have once again been picked for the analysis from Fig. 2. (a) *t* = 0.5 μm, (b) *t* = 2 μm.

As it is depicted in Fig. S5, the thicker shell's SCS is getting saturated much more quickly at lower σ_{VO2} values, which leaves a shorter range of exploration for the proposed device as opposed to the thinner shell configurations with less sensitivity and thereby with the added larger range of tunability. These results are already consistent with our earlier assessments in the previous sections regarding the thicker shell being more sensitive to

the variations. Hence, one must carefully plan to use the sensitivity for their advantage in order adapt the proposed device to sense minute changes in the temperature.

Figure S5 also suggests that the most exciting tunability range happens to be within 0-2e4 S/m. Higher values are not ideal as the mechanism becomes completely loss dominant. This point is critical as the proposed design is dependent on slight temperature changes. It is evident that huge temperature jumps do not give a larger range, in contrast they may have the chance of completely diminishing the working mechanism of the proposed structure.
